# *Plasmodium vivax *populations revisited: mitochondrial genomes of temperate strains in Asia suggest ancient population expansion

**DOI:** 10.1186/1471-2148-12-22

**Published:** 2012-02-17

**Authors:** Miao Miao, Zhaoqing Yang, Harland Patch, Yaming Huang, Ananias A Escalante, Liwang Cui

**Affiliations:** 1 Department of Entomology, Pennsylvania State University, University Park, PA 16802, USA; 2 Parasitology Department, Kunming Medical College, Kunming, Yunnan, China; 3 Malaria Department, Guangxi CDC, Nanning, Guangxi, China; 4 School of Life Sciences, Arizona State University, PO Box 874501, Tempe, AZ, USA

**Keywords:** genetic diversity, mitochondrial DNA, *Plasmodium vivax*, biogeography

## Abstract

**Background:**

*Plasmodium vivax *is the most widely distributed human malaria parasite outside of Africa, and its range extends well into the temperate zones. Previous studies provided evidence for vivax population differentiation, but temperate vivax parasites were not well represented in these analyses. Here we address this deficit by using complete mitochondrial (mt) genome sequences to elucidate the broad genetic diversity and population structure of *P. vivax *from temperate regions in East and Southeast Asia.

**Results:**

From the complete mtDNA sequences of 99 clinical samples collected in China, Myanmar and Korea, a total of 30 different haplotypes were identified from 26 polymorphic sites. Significant differentiation between different East and Southeast Asian parasite populations was observed except for the comparison between populations from Korea and southern China. Haplotype patterns and structure diversity analysis showed coexistence of two different groups in East Asia, which were genetically related to the Southeast Asian population and Myanmar population, respectively. The demographic history of *P. vivax*, examined using neutrality tests and mismatch distribution analyses, revealed population expansion events across the entire *P. vivax *range and the Myanmar population. Bayesian skyline analysis further supported the occurrence of ancient *P. vivax *population expansion.

**Conclusions:**

This study provided further resolution of the population structure and evolution of *P. vivax*, especially in temperate/warm-temperate endemic areas of Asia. The results revealed divergence of the *P. vivax *populations in temperate regions of China and Korea from other populations. Multiple analyses confirmed ancient population expansion of this parasite. The extensive genetic diversity of the *P. vivax *populations is consistent with phenotypic plasticity of the parasites, which has implications for malaria control.

## Background

*Plasmodium vivax *malaria threatens 40% of the world's population and causes over 132 million clinical cases every year [1]. Compared with other human malaria parasites, *P. vivax *has the widest global distribution and occurs in Asia, the Middle East, South and Central America, and parts of Africa [2]. Contrary to its common name "benign tertian malaria", *P. vivax *is also associated with severe pathology [3]. Furthermore, infection of Duffy-negative patients in Madagascar suggests that this parasite is evolving to exploit alternative invasion pathways, posing new threats to previously resistant human populations [4]. Yet, vivax malaria has been neglected, and the first-line treatments for vivax malaria have remained unchanged for 50 years. As malaria elimination and eradication are once again on the agenda of many malaria-endemic nations, *P. vivax *research and control is receiving renewed attention.

The history of malaria control provides considerable evidence for the resilience of *P. vivax *malaria and the underlying genetic diversity of the parasite. Indeed, across its geographic range, *P. vivax *displays extraordinary phenotypic variation in disease pathology, vectorial preference, and the pattern and frequency of relapse in the host (the time period at which the parasite remains dormant in the liver) [5]. To date, the processes which have led to the diversification of *P. vivax *are still not well understood.

Most investigations of the evolutionary history and population structure of *P. vivax *have relied on molecular markers. In an early study, low genetic variability at 13 microsatellite loci suggested little differentiation and a recent (< 10,000 years) world expansion of this parasite [6]. However, several later studies refuted this notion and suggest that Leclerc et al. [6] used a biased set of microsatellite markers [7,8]. Two recent comparisons of sympatric parasite populations further suggested that *P. vivax *microsatellites are more polymorphic than those of *P. falciparum *[9,10]. Other recent molecular phylogenetic analyses support an ancient demographic history of the *P. vivax *population, probably as a result of host switch from Asian monkeys [11-14]. While such an opinion is often inferred from the high level of genetic diversity in the parasite in Asia [15] and may not reflect the true origin of this parasite, it is certain that significant subdivision of the parasite populations exists [7,14,16]. Whereas most studies agree that the oldest populations appear to have originated in Southeast Asia [12-14], it is also true that the sampling has been opportunistic with poorly defined populations, making it difficult to properly assess the parasite demographic history [14].

The geographic range of *P. vivax *extends well into the temperate zone. The ability to survive in cool climates is attributed to sporogony in mosquitoes at a much lower temperature and the production of long-term hibernating hypnozoites in human liver. The latter adaptation is especially important for temperate strains, where the primary infection occurs eight months or more following inoculation by an infected mosquito [17]. In recent years, there has been a reemergence of vivax malaria in central China and on the Korean peninsula in areas where malaria control was highly successful during the global malaria eradication campaign of the 1960s [18,19]. The process of population expansion and spread of temperate strains is not understood. Although earlier evolution and population studies provided evidence about population differentiation of these Asian temperate vivax parasites [12,13,20], such analyses would benefit greatly from more extensive and spatially-defined sampling of these populations.

**Figure 1:**
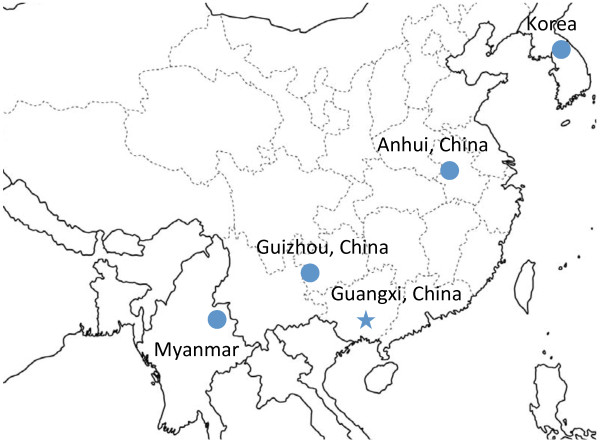
**Distribution of *P. vivax *sampling sites**. Circles indicate sampling sites in the present study, while the star marks the location of Guangxi province (a possible earlier sampling site).

Being genome-level informative, mitochondrial (mt) DNA sequences have been used for population and phylogenetic studies in a wide range of organisms [12,13,21-23]. In order to further define the demographic history and population structure of *P. vivax *in East and Southeast Asia, we sampled four *P. vivax *populations in a transect across temperate (Korea and central China) and warm temperate (southwestern China and northeastern Myanmar) climates (Figure 1). We obtained sequences of the complete mt genome from 99 parasite isolates. Genetic data were analyzed to look for evidence of genetic structure throughout the range of this parasite in temperate zone. The demographic histories of these populations were inferred in order to illuminate how this parasite species has evolved.

## Methods

### Sample collection, DNA extraction, PCR amplification and DNA sequencing

A total of 99 clinical *P. vivax *parasite samples were previously collected in four locations of East and Southeast Asia, including 36 samples from Bengbu city, Anhui Province of China in 2004, 26 samples from Luodian county, Guizhou Province of China in 2005, five samples from villages near the northern border of South Korea in 2005, and 32 samples from Laiza township of northeastern Myanmar in 2006-2008 (Figure 1) [20,24,25]. Collection sites in China and Korea were malaria hypoendemic with seasonal occurrence of only *P. vivax *parasites, whereas the Myanmar site was mesoendemic with perennial circulation of both *P. vivax *and *P. falciparum *parasites [26]. Ethical clearance was obtained from provincial ethical review committees of Guangxi and Anhui, and Kunming Medical University Institutional Review Board, respectively. The samples used for sequencing have been identified as single-clone infections by genotyping the *merozoite surface protein *(*msp*) *3α *and *msp3β *genes [20,25]. The highly polymorphic nature of these two genes should allow us to exclude most of the mixed-clone infections. Parasite DNA was extracted from blood filters using the QIAamp DNA Mini Kit (Qiagen, USA) with minor modifications. Primers were designed to amplify the complete *P*. *vivax *mt genome based on the sequence of the reference strain Sal-I (GenBank: AY598140). Long-range, high-fidelity PCR amplification was performed using Advantage HD DNA Polymerase Mix (Clontech, Mountain View, CA), which has efficient 3' → 5' exonuclease proof-reading activity. PCR reactions in 50 μl contained DNA template, 1 μM of each oligonucleotide primer, 10 × Advantage HD PCR Buffer, 1 mM deoxynucleosides (dNTPs), and 5 units of polymerase mix. PCR was performed at 94°C for 1 min, followed by 30 cycles of 94°C for 1 min, 60°C for 2 min, and 72°C for 3 min. A final extension was done at 72°C for 10 min. PCR products were purified with the PrepEase Gel Extraction Kit (USB, Affymetrix, USA) and sequenced using the BigDye version 1.1 on an ABI 3730XL sequencer. Overlapping sequences were obtained with the primers listed in Additional file 1. Sequences were base-called and assembled with PHRED and PHRAP [27,28], and heterozygous bases were called with the Polyphred program [29] and confirmed by visual inspection of the corresponding trace files using the CONSED trace file viewer [30]. To remove potential nucleotide ambiguities, certain sequences were confirmed by two independent PCR reactions from the same DNA template. The sequenced fragments were assembled into complete mt sequences and deposited in GenBank (JQ240331-JQ240429).

### Sequence alignment and haplotype network

For phylogenetic analysis, we retrieved additional 291 complete *P. vivax *mt genome sequences from GenBank (AY791517-AY791692, AY598035-AY598140, and AB550270-AB550280). Nucleotide diversity (*π*) - the average number of nucleotide differences per site between two sequences, and haplotype diversity (*h*) - the number and frequency of different haplotypes in the samples were estimated using DnaSP version 5.10 [31]. The three protein-coding genes were identified through alignment with annotated genes from the published *P. vivax *mt genomes. Sequence alignments were performed using inGAP v 2.2 [32] and ClustalW [33], and were manually checked using BioEdit http://www.mbio.ncsu.edu/BioEdit/bioedit.html. To detect selection acting on these coding sequences, the number of synonymous nucleotide substitutions per synonymous site (*d_S_*) and number of nonsynonymous nucleotide substitutions per nonsynonymous site (*d_N_*) were calculated using the SNAP program http://www.hiv.lanl.gov/content/sequence/SNAP/SNAP.html. Phylogenetic trees were constructed by using the maximum likelihood method implemented in BioNumerics v5.10 (Applied-Maths, Sint Maartens-Latem, Belgium). In order to define the relationship among the isolates at a micro-evolutionary level, we performed allelic profile-based comparison using a minimum spanning tree analysis with the TCS1.21 [34] as well as the BioNumerics v5.10 software. In TCS, inferences depend on the chosen probability of parsimony, for which we tried a value of 91%-95%. The number of mutational differences associated with the probability just before the 91% cutoff is the maximum number of connections between sequences. Ambiguities were treated according to the previous criteria [35].

### Population structure

Analysis of population structure was performed using STRUCTURE [36], which uses a Bayesian approach to calculate the posterior probability of the number of populations in the sample single nucleotide polymorphism (SNP) set. Replicate runs of STRUCTURE used a burn-in period of 50,000 iterations followed by a Markov chain Monte Carlo (MCMC) 100,000 iterations from which estimates were obtained. All runs were based on the admixture model without prior population information [36]. Twenty replicate unsupervised runs were performed for each value of the number of clusters *K *from 1 to 14. The number of population clusters was inferred according to Evanno et al. [37] and the *ad hoc *statistic ΔK was calculated for the presence of individuals with ancestry in two or more of the *K *populations. Graphs of the STRUCTURE results were made using DISTRUCT. Sequences were analyzed for maximum sequence diversity and visually inspected with highlighter tools http://www.hiv.lanl.gov.

ARLEQUIN software package version 3.11 was used to estimate genetic diversity indices and to assess population differentiation [38]. Pairwise comparisons *F*_ST _and *Φ*_ST _values were estimated by permutation analyses using 1,000 permutations with an assumption of no difference between populations. The *P*-value was considered as the proportion of permutations resulting in the higher *F*_ST _or *Φ*_ST _value or equal to the observed one. Analysis of molecular variance (AMOVA) was used to evaluate the extent to which sequence variation was partitioned among populations and areas.

### Neutrality tests and historical expansions

Neutrality statistics and pairwise nucleotide differences were calculated to examine the historical demographic expansions of *P. vivax*. Tajima's D test [39] and Fu's *Fs *test [40] were used to test whether the mt genome data conform to the expectations of neutrality. Tajima's D test compares two estimators of the mutation parameter θ, Watterson's estimator θs and Tajima's estimator θπ, and significant D values can be due to factors such as population expansion, bottlenecks and selection [39]. Fu's *F*s test compares the number of haplotypes observed with the number of haplotypes expected in a random sample under the assumption of an infinite-sites model without recombination, and *F*s was sensitive to population demographic expansion [40]. In addition, historic demographic expansions were also investigated by examining frequency distributions of pairwise differences between sequences (mismatch distribution), which is based on three parameters: θ_0_, θ_1 _(θ before and after the population growth) and τ (time since expansion expressed in units of mutational time) [41,42]. The expected mismatch distribution under a constant population size was estimated by the equation F*_i _*= θ^i^/(θ+1)^i+1^, where F*_i _*is the expected frequency of pairwise comparisons showing *i *differences, and θ is estimated by the observed mean of pairwise differences. The distribution usually has a ragged profile at demographic equilibrium due to stochastic lineage loss, but it is usually unimodal in populations following a recent population demographic expansion and population range expansion [41,43]. This reflects an underlying star-like genealogy in which all of the coalescent events occurred in a narrow time window. Both mismatch analysis and neutrality tests were performed in ARLEQUIN.

### Estimating the most recent common ancestor (TMRCA)

TMRCA of *P. vivax *was estimated using the strict-clock Bayesian MCMC method as implemented in BEAST v1.5.4 [44] with non-coding region of the mitochondrial genome. We assumed a general time-reversible model of nucleotide substitution with gamma-distributed rate heterogeneity among sites and a proportion of invariant sites. In addition, we assumed an uncorrected log normal distribution molecular clock model of rate variation among branches in the tree. As previously described, TMRCA was calculated using the divergence between *Plasmodium fragile *and *Plasmodium knowlesi*, assuming that they diverged as part of the radiation of their hosts (the *silenus *group of macaques and the divergence of the *sinica *and *fascicularis *groups) between 3.5 and 4.7 million years ago [14]. Analyses were performed for 1,000,000 generations with the model of Yule Process and Bayesian skyline analysis. The initial 20% of each run was discarded. The effective sample size for parameter estimates and convergence was checked using Tracer http://beast.bio.ed.ac.uk/Tracer.

## Results

### Genetic variation in *P. vivax *mt genomes from Myanmar, China and Korea

We sequenced the complete mt genomes from 99 *P. vivax *samples representing four populations collected in temperate and warm temperate regions of East Asia and Myanmar (Figure 1 and Table 1). Alignment of these complete mt genomes identified 26 SNPs (indels excluded), including 15 transversions and 11 transitions (Additional file 2). Sixteen of 26 SNPs are located in the three coding regions (COX3, COX1 and CYTB). Nucleotide diversity ranged from 0.00020 for China's Anhui population to 0.00096 for the combined Korean population. The *d*_N_/*d*_S _ratios of coding regions from all geographical sample sets were well below one, confirming that purifying selection is the predominant force in mtDNA evolution (Table 1). The 26 SNPs defined a total of 30 mt genome haplotypes. Compared with the number of haplotypes/population (8-38) identified in earlier studies, the numbers of haplotypes in our studied populations were modest, being the lowest (5) in China's Anhui population and highest (16) in the Myanmar population (Table 1). Likewise, haplotype diversity also varied, ranging from 0.584 in the Anhui population to 0.850 in the Myanmar population. The two temperate populations from China had much lower haplotype diversity and were comparable with the New World parasite population (0.58-0.68).

**Table 1:** Summary of molecular diversity for all sampled *P. vivax *populations ^a^

Geographic origin	Sample size	Date of collection	No. of haplotypes	No. of unique haplotypes	*S*	*h*	*π*	*k*	*d*_S_	*d*_N_
Myanmar	32	2006-2008	16	15	18	0.850 ± 0.057	0.00035 ± 0.00007	2.115 ± 1.464	0.0020	0.0005
Anhui, China	36	2004	9	7	14	0.584 ± 0.093	0.00020 ± 0.00006	1.211 ± 0.625	0.0014	0.0010
Guizhou, China	26	2005	5	3	7	0.683 ± 0.068	0.00043 ± 0.00008	2.548 ± 2.000	0.0014	0.0012
South Korea ^b^	17	-	9	4	19	0.875 ± 0.058	0.00096 ± 0.00011	5.721 ± 8.309	0.0024	0.0016
China ^c^	34	-	11	9	15	0.875 ± 0.036	0.00063 ± 0.00006	3.774 ± 3.801	0.0021	0.0010
South America	47	-	9	5	9	0.642 ± 0.071	0.00019 ± 0.00003	1.164 ± 0.584	0.0026	0.0004
Africa	12	-	8	6	15	0.894 ± 0.078	0.00046 ± 0.00012	2.742 ± 2.440	0.0022	0.0006
South and west Asia	35	-	17	12	25	0.884 ± 0.043	0.00047 ± 0.00009	2.800 ± 2.296	0.0026	0.0009
Southeast Asia ^d^	56	-	30	24	39	0.946 ± 0.019	0.00058 ± 0.00005	3.471 ± 3.231	0.0025	0.0007
Indonesia	22	-	13	10	17	0.840 ± 0.078	0.00029 ± 0.00007	1.710 ± 1.077	0.0016	0.0007
Melanesia	73	-	38	30	44	0.931 ± 0.019	0.00056 ± 0.00006	3.355 ± 3.029	0.0027	0.0006

^a^Shown are number of segregating sites (*S*), haplotype diversity (*h *± standard deviation), nucleotide diversity (*π *± standard deviation), average pairwise difference among individuals (*k *± total variance), the average number of nucleotide substitutions per non-synonymous site (*d*_N_) and synonymous sites (*d*_S_) for each grouping of samples, calculated based on the mitochondrial DNA sequences. ^b ^Korean sequences included those from reference [43] and the present study.

^c ^Additional sequences from China were obtained from references [12] and [13].

^d ^Samples from Thailand and Vietnam were grouped within Southeast Asia.

### Phylogeography

**Figure 2:**
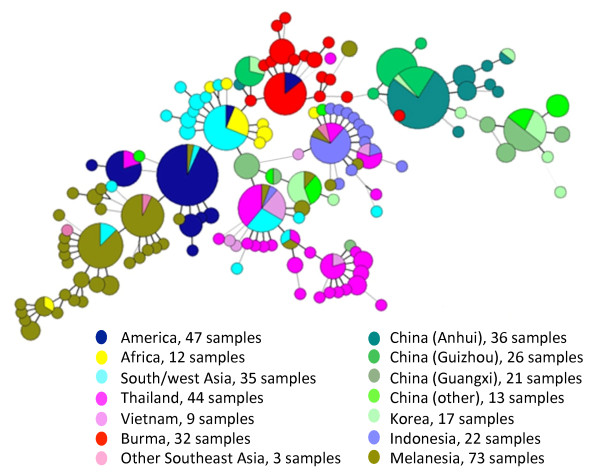
**A haplotype network based on *P. vivax *mt genome sequences**. Each circle represents a haplotype with size of the circle equaling to the frequency of the haplotype in the samples. Lines separating haplotypes represent mutational steps. Each color shows the countries of origin of the samples.

Analysis of *P. vivax *mt genomes from 99 newly collected Asian samples as well as 291 previously described isolates from 11 populations identified a total of 141 haplotypes [12,13,45]. Most geographical regions had one predominant haplotype, whereas Melanesia, China, and Southeast Asia had two predominant haplotypes. To characterize the frequencies and relationships of different haplotypes, a minimum spanning tree was constructed. This haplotype network clearly showed geographical clustering of the haplotypes (Figure 2). Inclusion of the temperate *P. vivax *samples did not dramatically change the overall topology of the network. Consistently, significant sharing of the haplotypes was detected between South/West Asian and African populations [12]. For the four temperate populations, most haplotypes were connected to the South/West Asian populations, which is consistent with the fact that these geographic regions are physically connected and past genetic exchanges might have occurred between these populations. Interestingly, the two temperate *P. vivax *populations from China's Anhui and Guizhou provinces formed a local network consisting of two predominant, related haplotypes. In contrast, the Chinese samples analyzed earlier (some from Guangxi Province in southern China, Figure 1) [12,13] formed two clusters, which might indicate discrete origins: some haplotypes were clustered in the East Asian branch, whereas others were shared and linked with haplotypes from Southeast Asia (Thailand, Vietnam and Indonesia). As reported previously [45], most Korean haplotypes were shared with those from southern China [12,13], but diverged from the central China populations.

To further illustrate the relationship of the mt genome haplotypes, a maximum likelihood phylogenetic tree based on the complete genome sequences was constructed (Additional file 3). All mt genomes from *P. vivax *were closely related to one another; no branches within the *P. vivax *cluster received strong bootstrap support (data not shown). In general, the topology of this tree roughly mirrored that of the minimum spanning tree. Both analyses showed that East Asian populations exhibited relatively large divergence from the remaining populations, suggesting that the temperate parasite populations in East Asia had a different demographic history.

### Population Structure

**Figure 3:**
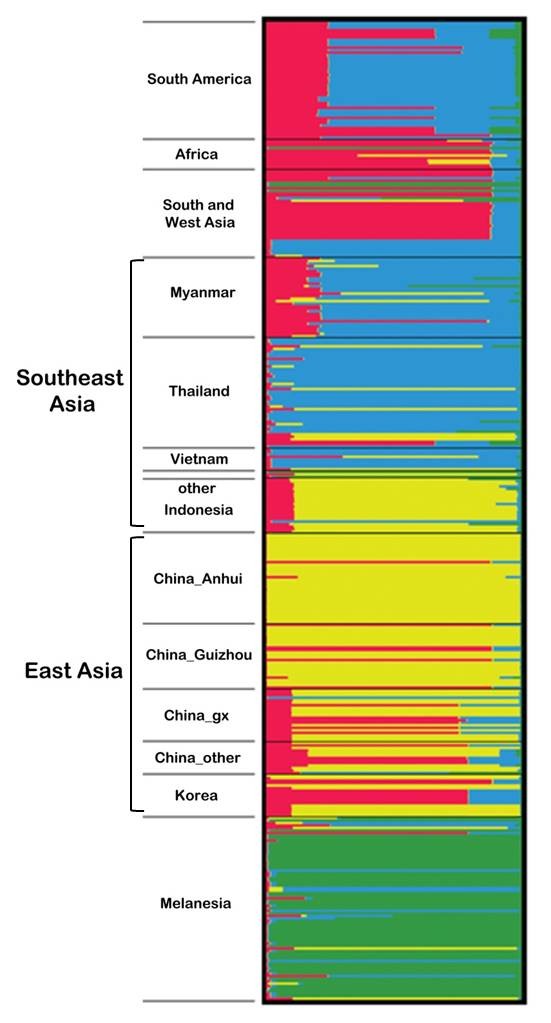
**Graphical display of estimated population structure at *K *= 4**. Each vertical line represents one individual. At *K *= 4, the samples are partitioned into four populations, which are consistent with large geographical regions of Melanesia, East Asia, Southeast Asia and the rest of the world. Populations are separated by black lines and occurred in the same order as given in Table 2.

Earlier studies using both mt genome and microsatellites have found significant differentiation of the extant *P. vivax *populations [7,12]. To further define genetic structure of the *P. vivax *populations, we used a Bayesian admixture procedure implemented in STRUCTURE to calculate the potential number of populations. Using a series of *K *values from 1 to 14, we detected a large incremental increase of the likelihood value as the number of genetic clusters in the model increased from 1 to 4 (data not shown). Significant genetic structure was found between parasite populations, and most parasites formed geographical clusters. At *K *= 2, only the East Asian cluster was separated from the rest of the populations, although this division was also supported from analysis of the distribution of *ΔK *using the method of Evanno et al. [37]. Posterior probability of the entire sample set at 4 populations was most consistent with a clear separation of populations according to large geographical regions of Melanesia, East Asia, Southeast Asia and the rest of the samples (Figure 3). It is noteworthy that South American and Myanmar populations appeared to be composed of a mixture of haplotypes from South/West Asia as well as Southeast Asia, suggestive of population boundaries or shared demographic histories of the parasite populations in these regions.

To further determine whether population differentiation exists, we estimated *F*_ST _(only based on haplotype frequency) and *Φ*_ST _(based on both haplotype frequency and genetic distance) for each population using the mtDNA sequences. The majority of pairwise *F*_ST _values were greater than 0.25 (ranging from 0.25 to 0.85), suggesting that strong population differentiation exists among the populations. Notable exceptions included the following pairwise comparisons: South/West Asia and Africa, South/West Asia and South America, South/West Asia and Southeast Asia, as well as Korea and southern China (Table 2). In Southeast Asia, parasite populations from the Greater Mekong subregion, Indonesia and Melanesia were also distinct with *F*_ST _values of 0.32 - 0.62 and Φ_ST _values of 1.58 - 4.84 (*P *= 0.01). For the temperate parasite populations from Asia, which include northeast Myanmar, Korean, and three Chinese *P. vivax *populations, between-population genetic differences were strong (*F*_ST _= 0.32-0.75, *Φ*_ST _= 0.97-5.13) and statistically significant after sequential Bonferroni correction (*P *< 0.0001). The only exception was between the Korean and southern Chinese population (exact origin of the samples unknown), which showed little genetic differentiation (*F*_ST _= 0.05, *P *= 0.08; *Φ*_ST _= 0.20, *P *= 0.08) (Table 2). We further grouped all studied populations into eight geographical groups consisting of Africa, America, South/West Asia, Southeast Asia, Myanmar, East Asia, Indonesia, and Melanesia, and performed a hierarchical AMOVA analysis. The three covariance components (within population, among population/within group, and among groups) could explain 22.7%, 32.3% and 45.0% of the variance, respectively. Apparently, considerable variation was preserved at the population level.

**Table 2:** Pairwise *F*_ST _(below diagonal) and *Φ*_ST _(above diagonal) of worldwide *P. vivax *populations

	1	2	3	4	5	6	7	8	9	10	11
**1**	-	0.9118	0.5650	1.0900	1.0042	4.5147	6.7287	4.8307	3.5576	3.0435	1.0581
**2**	0.3953	-	0.0836	1.7741	1.2535	2.6387	5.0053	3.1483	2.0086	1.6117	1.4914
**3**	0.2346	0.0285	-	1.4638	0.7635	3.0290	5.7006	3.8684	2.1737	1.8908	1.1488
**4**	0.4140	0.4395	0.3711	-	1.7561	5.1579	5.1297	3.3530	4.0720	3.2075	1.9961
**5**	0.2920	0.2680	0.1908	0.3689	-	2.5679	6.2880	4.6245	1.7653	1.9738	1.5809
**6**	0.7718	0.5624	0.5583	0.7250	0.4596	-	3.7527	2.8901	0.9766	1.4396	4.8403
**7**	0.8501	0.7616	0.7404	0.7579	0.7073	0.7284	-	0.9711	2.7673	2.1942	7.2903
**8**	0.7454	0.5468	0.5889	0.5918	0.5913	0.5709	0.3557	-	2.4518	1.6907	5.4439
**9**	0.6123	0.3597	0.3980	0.5775	0.3297	0.2981	0.5295	0.4292	-	0.1958	4.0072
**10**	0.5716	0.2582	0.3408	0.4929	0.3363	0.2437	0.4625	0.3122	0.0485	-	3.6619
**11**	0.2952	0.3094	0.2644	0.3991	0.3166	0.6168	0.7323	0.6327	0.5343	0.4947	-

The geographical origin of each population is indicated as: 1 - South America, 2 - Africa, 3 - South and West Asia, 4 - Myanmar, 5 - Southeast Asia, 6 - Indonesia, 7 - China (Anhui), 8 - China (Guizhou), 9 - China (other), 10 - Korea, 11 - Melanesia.

### Demographic history

**Figure 4:**
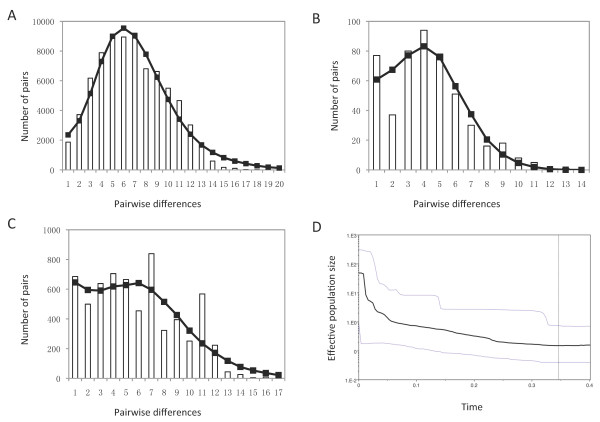
**Historical demographic history of *P. vivax *inferred from whole mt genome**. **A**. Pairwise mismatch distributions for worldwide *P*. *vivax*. Observed distributions (columns) were compared with the expected values for populations that have undergone historical demographic expansion (lines and circles). **B**. Pairwise mismatch distributions for *P. vivax *from Myanmar. **C**. Pairwise mismatch distributions for vivax species from East Asia. **D**. Bayesian skyline plots for the same regional groups with A. Black lines represent median population estimates and grey lines denote upper and lower confidence limits (95% highest posterior density).

The star-like shape of the haplotype network is consistent with significant population expansion (Figure 2). To test this hypothesis, we performed mismatch distribution and skyline analyses. Mismatch distribution was unimodal for the entire *P. vivax *sample set (Figure 4A), a pattern characteristic of populations that have undergone large-scale expansion. To obtain more precise estimates for the temperate parasite populations, we divided our recent samples into two large groups represented by the Myanmar and East Asian populations (Chinese and Korean samples combined), and performed neutrality tests and mismatch distribution analysis. Although mismatch distributions for these two populations did not perfectly fit the expected pattern under the sudden expansion model (Figure 4B and 4C), they did not differ significantly (*P *> 0.05) from this model and therefore were suitable for the analysis of demographic patterns. The *F*s test and Tajima's *D *test agreed well with the mismatch analyses. The *F*s values of the entire *P. vivax *populations as well as the East Asian and Myanmar populations were negative and significant (*P *= 0.025 for East Asian populations and *P *= 0.005 for Myanmar population). Tajima's *D *test compares two mutation estimators *θ*s and θπ, which are affected by the size of the present-day and original population, respectively [39]. Consequently, a history of population growth would inflate *S *relative to *π *and generate a negative value of *D *[46,47]. Consistent with a past population expansion, Tajima's *D *test was negative (-2.05) and statistically significant (*P *= 0.001) for the entire *P. vivax *populations analyzed. For the Myanmar and East Asian populations, the *D *statistic was negative and only significant for the Myanmar population (*P *= 0.021). The τ value, which reflects the location of the mismatch distribution crest, provides a rough estimate of the time when rapid population expansion started. The observed value of the age expansion parameter (τ) was 4.18 units (95% CI: 2.56-10.41) of mutational time in *P. vivax*. In comparison, the τ value of the Myanmar population was smaller (4.10, 95% CI: 1.36- 6.59). Yet, the East Asian populations had a τ value of 5.13 (95% CI: 2.01- 13.3), which was even greater than that for all vivax populations, suggesting that the East Asian parasite populations may be at demographic equilibrium in recent evolutionary history. The results of the skyline analysis further supported *P. vivax *population expansion showing a rapid increase in effective population size starting ~ 200,000 years before present (ybp), followed by a more recent increase in population numbers starting at ~50,000 ybp (Figure 4D).

We estimated the divergence time of Myanmar *P. vivax *population to be 385,000 (± 3,800) years ago, whereas the East Asian *P. vivax *population diverged at 383,000 (± 7,300) years ago. Our estimate also indicated that the TMRCA for the whole vivax populations was around 346,000 to 452,400 years ago (95% highest posterior density).

## Discussion

The demographic history and genetic diversity of *P. vivax *provide a foundation for the development of effective malaria control measures. Phylogenetic analysis of *P. vivax *using sequences from the mt genome and nuclear genes has placed this parasite in a clade that includes the Asian monkey parasites *P. cynomolgi*, *P. simiovale *and *P. knowlesi *[11-13]. This phylogeny is consistent with an Asian origin of *P. vivax*, suggesting this parasite is probably the descendent of an ancestor that switched from Asian monkeys to humans. Regardless of what calibration points were used, TMRCA estimates support an ancient demographic history of the extant *P. vivax *parasites [12-14,48], which concurs with the genetic diversity of global *P. vivax *strains. *P. vivax *parasites also display significant phenotypic diversity such as different relapse patterns, which serve to distinguish tropical from temperate strains. Temperate strains with primary infection occurring eight months or more following inoculation by an infected mosquito were proposed to be a subspecies of *P. vivax *[49]. Similarly, New World *P. vivax *parasites were postulated to represent a separate subspecies based on molecular polymorphism and difference in vector preference [50], albeit such a hypothesis was not supported by the mt haplotype analysis [12]. Therefore, more detailed sampling of *P. vivax *populations from its entire geographical range is necessary to better understand the evolutionary history of *P. vivax*.

Extant *P. vivax *populations may have been influenced by historical population expansions and more recent migrations. In addition, the evolutionary process, often estimated from the geographical pattern of genetic variation, can be influenced by colonization events such as range expansion or reduction [51,52]. Several lines of evidence support the hypothesis of ancient population expansion of *P. vivax*. First, the existence of a phylogeny consisting of several distinct, but closely related lineages suggests a rapid population expansion in the recent past (Figure 2). Second, we found an extremely close fit between the observed pairwise mismatch distribution and the expected distribution based on a model of rapid population expansion (Figure 4). Finally, the skyline plot shows a period of a more recent increase in population size. This period is concordant with a time of human migration. *Homo sapiens *had occupied Africa about 150,000 Mya. They moved out of Africa 70,000 years ago and spread into Asia, Europe and Australia 40,000 years ago. It is also possible that the population expansion of *P. vivax *was linked to the expansion not only in the human host but also in adaptation of the mosquito vectors. However, it needs to be cautioned that mtDNA has a smaller effective population size and provides only part of a species' history; thus more robust data from nuclear genes are needed to corroborate this conclusion [53,54].

Despite generally low endemicity, the global *P. vivax *populations display high genetic diversity at microsatellite, SNP, and antigenic loci [5,7,15,55-57]. Previous studies of *P. vivax *parasites from Myanmar and central China demonstrated high genetic diversity and multiple-clone infections [20,55,58]. Our analysis of the mt genome also detected comparable, high-level genetic diversity among these temperate *P. vivax *populations. Consistent with high malaria endemicity in Myanmar, haplotype diversity was also high (0.85 ± 0.057) and comparable to other highly endemic areas of the world [12]. However, haplotype diversity of the two temperate populations from China was lower and at similar levels to those of the New World parasite populations. This result appeared to be consistent with the recent history of malaria epidemiology in central China. Historically, temperate *P. vivax *malaria was highly prevalent in central China, but it was considerably curtailed during the global malaria eradication campaign in the 1950s and 1960s [59]. However, in the last two decades *P. vivax *malaria has resurged and outbreaks occurred in several central provinces [18,60]. We thus speculate that past strenuous control efforts might have caused a population bottleneck in the parasite population and as a result the diversity of resurging parasites was reduced. This bottleneck effect on *P. vivax *population was also found in southern Thailand, where *P. vivax *population displayed a high level of clonality [61].

For a finite population, unless there is complete panmixia and random sampling, a pattern of genetic isolation by geographic distance is generally expected [62]. This principle applies well to the *P. vivax *populations. Within its geographic range, *P. vivax *exhibits substantial population differentiation, especially between different continents [7,12]. The clear differentiation between parasites from Melanesia and those from Southeast Asian countries is much surprising, since previous microsatellite-based analyses of both *P. vivax *[8] and *P. falciparum *[63] failed to show such a clear pattern. *F*_ST _and *Φ*_ST _statistics revealed significant population differentiation between Myanmar and East Asian *P. vivax *populations. Even within a short distance, genetic differentiation may be significant due to possible migration or ecological constraints [64]. Substantial genetic structure existed between the Chinese Guizhou and Anhui *P. vivax *populations despite their geographic proximity. Interestingly, two major genotypes in China were also observed in the South Korean population [45,65]. Population genetic structure can result from both species-specific biological traits and abiotic factors. The temperate populations of *P. vivax *have developed a trait of long relapsing liver hypnozoites, an adaptation to the long winter period of temperate climate when transmission is interrupted [66]. Because of the obligatory role of mosquito vectors in malaria transmission, reciprocal selection between malaria parasites and mosquito vectors can lead to local adaptation of the parasite [16]. It is unknown whether vector adaptation plays any role in the population structure of these temperate parasites. Also, genetic drift acting on small populations (from areas of low endemicity) may be a force driving population differentiation [7,9,67].

Since genetic diversity of the global *P. vivax *populations has been suggested to be the result of ancient hominid geographical expansion [13], the relationships among the extant parasite populations might reflect past demographic histories of the parasites and the routes by which parasite populations have expanded. Most mtDNA haplotypes from the four temperate and warm temperate populations were unique but related, suggesting that they might be descendents from the same lineage(s). Haplotype network analysis suggested South/West Asia as the root or origin of the parasite populations (Figure 2), but this conjecture does not exclude a possible African origin of *P. vivax*, as African parasites shared the major mtDNA haplotype with the South/West Asian samples. Culleton and collegues proposed that the present-day African and American populations may be the closest extant relatives of the African ancestor [68]. Since clustering in the network is often affected by the methodologies used, the exact origin of the vivax ancestor is still not clear. Haplotype network analysis also showed that samples collected in China formed two divergent lineages: one (possibly from subtropical southern China) was closely related to the Southeast Asian samples (Indonesia, Thailand, and Vietnam), whereas the other (mostly temperate strains) was directly diverged from the northeast Myanmar population. Myanmar is connected to East Asia, Southeast Asia, and South/West Asia and such a geographical location may be critical for elucidating the population expansion and evolutionary history of *P. vivax*. The relationship of the temperate Chinese and northeast Myanmar *P. vivax *populations points to a possibility of population expansion from South/West Asia to temperate China via northeast Myanmar, which seems to make sense from a geographic point of view. It is noteworthy that the *P. vivax *parasites from northeast Myanmar, China and Korea all have similar, long relapsing patterns characteristic of temperate *P. vivax *strains [69]. Furthermore, our results are consistent with the notion that temperate and warm temperate *P. vivax *parasites may represent a unique lineage, which is important to elucidate the genetic structure and history of expansion of *P. vivax*.

## Conclusion

The present study readdressed the issue of the extant *P. vivax *population structure by focusing on temperate zone parasite populations in East and Southeast Asia. Analysis of the complete mt genomes from 99 clinical samples confirmed that *P. vivax *displays extensive genetic diversity and natural populations are clearly structured. While most mtDNA haplotypes from the four temperate populations were related, suggestive of descendent from the same lineage(s), local population subdivision was also apparent. Multiple tests further confirm the ancient expansion of the *P. vivax *population.

## Abbreviations

Mt: mitochondrial; *d_S_*: the number of synonymous nucleotide substitutions per synonymous site; *d_N_*: the number of nonsynonymous nucleotide substitutions per nonsynonymous site; Markov chain Monte Carlo (MCMC): TMRCA: the most recent common ancestor; SNP: single nucleotide polymorphism; ybp: years before present.

## Authors' contributions

LC designed experiments, guided the work and helped to draft the manuscript. MM carried out the molecular studies, analyzed data and drafted the manuscript. ZY provided the samples and carried some molecular studies. YH provided the samples. PH and AE revised the manuscript. All authors read and approved the final manuscript.

## Supplementary Material

Additional file 1 **Primer sequences**. Primers used for amplifying and sequencing the mitochondrial genome of *Plasmodium vivax*. Click here for file

Additional file 2 **Distribution of SNPs in the *Plasmodium vivax *mitochondrial genomes isolated from Myanmar, China and South Korea**. The graph shows the positions of the SNPs in the 5900 bp mt genome for the 30 haplotypes (h1 - h30) of *P. vivax *populations. Click here for file

Additional file 3 **Phylogeny of *Plasmodium vivax *parasites**. A maximum likelihood tree based on mitochondrial genomes of 390 *Plasmodium vivax *samples. Click here for file
