# Supplementary material for: Plasmodium vivax populations revisited: mitochondrial genomes of temperate strains in Asia suggest ancient population expansion
Source: BMC Evol Biol. 2012 Feb 17;12:22. doi: 10.1186/1471-2148-12-22 (PMC3305529; doi:10.1186/1471-2148-12-22)
Supplement: Additional file 1 — Primer sequences. Primers used for amplifying and sequencing the mitochondrial genome of Plasmodium vivax. [file 1471-2148-12-22-S1.DOC]

**Additional file 1: Primer sequences.** Primers used for amplifying and sequencing the mitochondrial genome of *Plasmodium vivax*.

| **Primer** | **Amplification /Sequencing** | **Sequence (5'-3')** |
| --- | --- | --- |
| PVMit301F | amplification/sequencing | CGCTGACTTCCTGGCTAAAC |
| PVMit3212R | amplification/sequencing | GTCAGGCGTTAAAAGCGTTC |
| PVMit2927F | amplification/sequencing | TTGTACACACCGCTCGTCAC |
| PVMit419R | amplification/sequencing | CCGAACCTTGGACTCTTGAA |
| PVMit1563F | sequencing | GGGAACAAACTGCCTCAAGA |
| PVMit1718R | sequencing | GCAAACACTAGCGGTGGAAT |
| PVMit3557F | sequencing | TGCCAGGATTATTTGGAGGA |
| PVMit5179R | sequencing | TTTGTCCCCAAGGTAAAACG |
| PVMit5910R | sequencing | AATGTTTGCTTGGGAGCTGT |
