# Supplementary material for: Plasmodium vivax populations revisited: mitochondrial genomes of temperate strains in Asia suggest ancient population expansion
Source: BMC Evol Biol. 2012 Feb 17;12:22. doi: 10.1186/1471-2148-12-22 (PMC3305529; doi:10.1186/1471-2148-12-22)
Supplement: Additional file 2 — Distribution of SNPs in the Plasmodium vivax mitochondrial genomes isolated from Myanmar, China and South Korea. The graph shows the positions of the SNPs in the 5900 bp mt genome for the 30 haplotypes (h1 - h30) of P. vivax populations. [file 1471-2148-12-22-S2.DOC]

**Additional file 2: Distribution of SNPs in the *Plasmodium vivax* mitochondrial genomes isolated from Myanmar, China and South Korea.** The graph shows the positions of the SNPs in the 5900 bp mt genome for the 30 haplotypes (h1 – h30) of *P. vivax* populations. A total of 26 SNPs were identified and depicted as vertical bars. Each vertical bar indicates a nucleotide difference from the reference sequence of the Sal-I strain (GenBank: AY598140). The bottom scheme illustrates the locations of the three coding regions, COX3, COX1 and CYTB.
