# Supplementary material for: Plasmodium vivax populations revisited: mitochondrial genomes of temperate strains in Asia suggest ancient population expansion
Source: BMC Evol Biol. 2012 Feb 17;12:22. doi: 10.1186/1471-2148-12-22 (PMC3305529; doi:10.1186/1471-2148-12-22)

**Additional file 3: Phylogeny of *Plasmodium vivax* parasites.** A maximum likelihood tree based on mitochondrial genomes of 390 *Plasmodium vivax* samples. The tree was constructed using GTR with a gamma shaped parameter of 0.55.


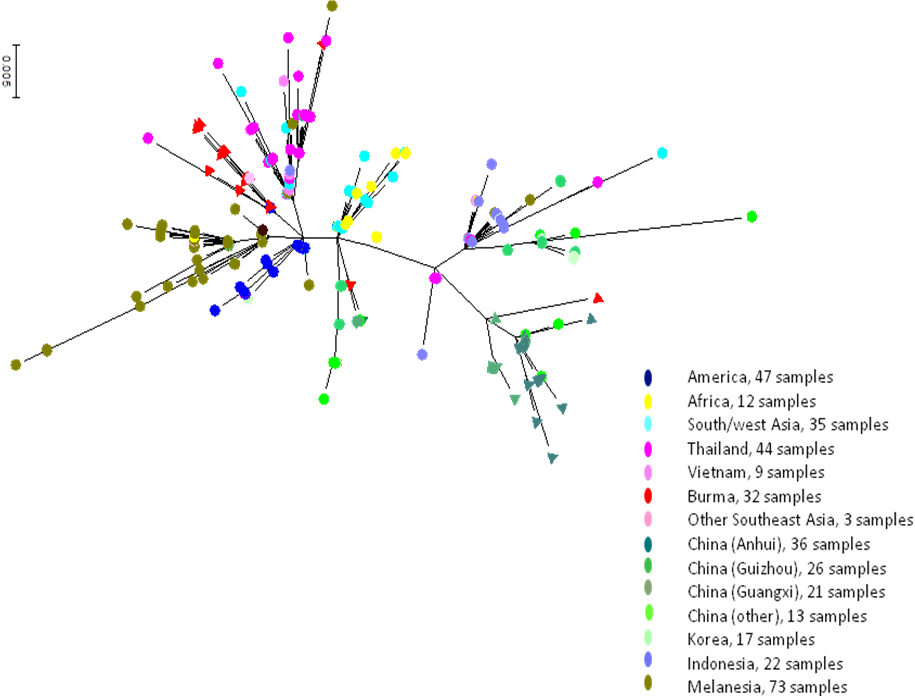

Supplement: Additional file 3 — Phylogeny of Plasmodium vivax parasites. A maximum likelihood tree based on mitochondrial genomes of 390 Plasmodium vivax samples. [file 1471-2148-12-22-S3.DOC]
